# Histone H3 Mutations: An Updated View of Their Role in Chromatin Deregulation and Cancer

**DOI:** 10.3390/cancers11050660

**Published:** 2019-05-13

**Authors:** Brandon R. Lowe, Lily A. Maxham, Joshua J. Hamey, Marc R. Wilkins, Janet F. Partridge

**Affiliations:** 1 Department of Pathology, St. Jude Children’s Research Hospital, 262 Danny Thomas Place, Memphis, TN 38112, USA; Brandon.Lowe@stjude.org (B.R.L.); Lily.Maxham@stjude.org (L.A.M.); 2 School of Biotechnology and Biomolecular Sciences, University of New South Wales, Kensington, NSW 2052, Australia; j.hamey@unsw.edu.au (J.J.H.); m.wilkins@unsw.edu.au (M.R.W.)

**Keywords:** histone H3.3, mutation, K27M, K36M, K9M, G34R, G34V, G34W, diffuse intrinsic pontine glioma, giant cell tumor of bone, chondroblastoma, chondrosarcoma, PRC2, SETD2

## Abstract

In this review, we describe the attributes of histone H3 mutants identified in cancer. H3 mutants were first identified in genes encoding H3.3, in pediatric high-grade glioma, and subsequently in chondrosarcomas and giant cell tumors of bone (GCTB) in adolescents. The most heavily studied are the lysine to methionine mutants K27M and K36M, which perturb the target site for specific lysine methyltransferases and dominantly perturb methylation of corresponding lysines in other histone H3 proteins. We discuss recent progress in defining the consequences of these mutations on chromatin, including a newly emerging view of the central importance of the disruption of H3K36 modification in many distinct K to M histone mutant cancers. We also review new work exploring the role of H3.3 G34 mutants identified in pediatric glioma and GCTB. G34 is not itself post-translationally modified, but G34 mutation impinges on the modification of H3K36. Here, we ask if G34R mutation generates a new site for methylation on the histone tail. Finally, we consider evidence indicating that histone mutations might be more widespread in cancer than previously thought, and if the perceived bias towards mutation of H3.3 is real or reflects the biology of tumors in which the histone mutants were first identified.

## 1. Introduction

Chromatin is composed of nucleosomes and their associated proteins, with each nucleosome consisting of an octamer of two copies each of histones H3, H4, H2A, and H2B, wrapped 1.7 times by 147 bp of DNA. Chromatin is critical for the control of transcription, replication, DNA repair and other aspects of genomic stability, including high fidelity chromosome segregation during cell division and maintenance of telomere integrity. Given its central role in the control of cellular processes, an exquisite regulatory system of chromatin biology has evolved. This relies heavily on post-translational modification (PTM) of chromatin, at both the DNA (methylation) and histone level, with an extensive array of modifications, including acetylation, methylation, phosphorylation, ubiquitination, sumoylation, and isomerization occurring on the histone tails that protrude beyond the nucleosomal DNA, as well as on the histone cores that are wrapped by the DNA.

Perhaps not surprisingly, the proteins that bind histones to “read” the code of modifications and to act upon those signals, are frequently mutated in cancer, causing a disruption of the cellular signaling pathways [1,2,3]. Mutations in chromatin-associated or regulatory proteins are widespread in pediatric cancers, and their prevalence in these frequently simple cancer genomes (implying that there are usually fewer mutations in pediatric cancers than in adult) highlights their importance in oncogenesis [4]. In 2012, genome sequencing efforts yielded the remarkable discovery that the histones themselves could be mutated in cancer. Two groups identified high-frequency somatic mutations in histone H3, in pediatric high-grade glioma (pHGG) [5,6]. Subsequent studies have extended the breadth of cancers known to carry mutations in H3 to include chondroblastoma, giant cell tumors of bone, chondrosarcoma, pediatric soft tissue sarcoma, head and neck squamous cell carcinoma and leukemia [7,8,9,10,11,12,13]. These mutants have generated substantial interest from both basic scientists and clinicians to discern how mutation of a single allele encoding H3 can exert dominant effects to alter H3 function in cells with 30 alleles encoding H3 isoforms, and how cells bearing these mutant H3s might be targeted for therapy. 

Here, we aim to highlight the areas of significant progress, since our initial review in 2015, of how histone H3 mutations might lead to chromatin deregulation in cancer [14]. We start by providing a short primer on histone H3 subtypes and refer the interested reader to our previous review for more information. We then review the current knowledge of the biological effects of the “K to M” mutant H3 class, where a lysine that is normally subject to methylation or acetylation is mutated to methionine, and the glycine 34 mutant class, where mutation of G34 impacts the modification of the nearby K36 residue.

### A Short Primer on Histone H3 Subtypes Relevant to This Review

In humans, there are different histone H3 variants. H3.3 proteins are expressed constitutively [15], while H3.1 and H3.2 are expressed only during the S-phase [16]. Some are expressed in a tissue restricted fashion (H3.5, H3.X, H3.Y) [17,18], or are deposited only at centromeres (CENP-A) [19].

Here, we will restrict the discussion to the most highly related histones H3.1, H3.2, and H3.3. H3.3 is encoded by two genes, *H3F3A* and *H3F3B*, H3.1 by ten genes, and H3.2 by three genes. While only a single amino acid change distinguishes H3.1 protein from H3.2, H3.3 differs by an additional four amino acid substitutions. These substitutions are mainly in the core of the histone protein, and dictate the binding specificity of the chaperone proteins, which associate with soluble histones and control their incorporation into and eviction from nucleosomes, contributing to the differential localization patterns of the H3 variants within the genome [20,21,22,23,24]. 

H3.3 is expressed throughout the cell cycle, as well as in quiescent cells [15], and specific chaperones deposit H3.3 into chromatin, independent of DNA replication. Histone cell cycle regulator (HIRA) deposits H3.3 at the euchromatic loci that become transiently depleted of nucleosomes, such as in the regions of high transcriptional activity [25,26,27,28,29,30]. Surprisingly, in addition to its deposition at very transcriptionally active regions of the genome, H3.3 is also deposited at silent or heterochromatic loci, including the pericentromeric and telomeric regions, by a distinct chaperone, the death-domain associated protein (DAXX) in complex with the transcriptional regulator α-thalassemia/mental retardation syndrome X-linked (ATRX) [22,23]. In contrast to the constitutively expressed H3.3, the DNA-replication-dependent canonical histone variants H3.1 and H3.2 are cell cycle regulated and deposited only during the S-phase and during DNA repair [16]. H3.1 is incorporated into nucleosomes by the chaperone CAF1 (chromatin assembly factor) [21,31,32], which is recruited to the newly synthesized DNA by its association with the replication factor, proliferating cell nuclear antigen (PCNA) [33]. Due to the differences in their expression and deposition, the levels of H3.1 and H3.3 vary widely in different cell types and tissues, and H3.3 can accrue to almost 90% of the total H3 in post-mitotic cells, such as neurons [34,35,36,37]. 

**Figure 1:**
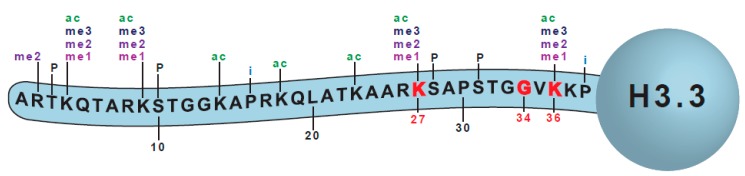
The amino terminal tail of histone H3.3. Commonly mutated sites are marked in red (K27, G34, K36) and the sites of modification are depicted as ‘me’—methylation, ‘ac’—acetylation, ‘P’—phosphorylation, and ‘i’—isomerization.

The first histone H3 mutations were identified in pHGG in 2012 [5,6]. Key features of the histone mutants are that they are somatic [6], and the mutations occur in only a single gene and arise at high frequency within a particular disease (Figure 1). Particular mutations occur in different tumors, in specific locations, and within a limited span of patient age (reviewed in [14]). These specific attributes have led to the mutant histones being coined “oncohistones”, and an acceptance that the mutants are ‘drivers’ of tumorigenesis [38].

## 2. The H3 K to M Mutants

### 2.1. K27M

#### 2.1.1. Cancer Association of K27M

The H3K27M mutation was first identified by genomic sequencing efforts in ~30% of pHGG, arising in the midline and affecting the thalamus, the basal ganglia, and the spinal cord [5,6,39]. The K27M mutation primarily occurs in *H3F3A* (>70%), one of the two genes encoding H3.3, with a low frequency occurrence in H3.1 encoded by *HIST1H3B* or *HIST1H3C*, and rarely in H3.2 *(HIST2H3C)* [5,6,40,41]. A particularly aggressive form of these tumors arises in the brain stem or the pons and is called Diffuse Intrinsic Pontine Glioma (DIPG). The K27M mutation is present in 80% of cases of DIPG, and these children have an expected survival rate of around 10% at 2 years following diagnosis [42]. In 2016, the world health organization classified K27M tumors as a distinct entity—“Diffuse midline glioma, H3K27M” [43]. The restriction of the K27M mutation to tumors arising in a certain region of the brain, suggests that K27M mutation might only provide a selective advantage for cell proliferation and transformation in a subset of cells, in a specific developmental context. This idea has been supported by several studies showing that a combination of drivers, including H3K27M, is necessary to drive tumorigenesis. Mutations in *TP53* and *PDGFRA* (platelet derived growth factor alpha) are often found to be associated with H3.3K27M in DIPGs, while H3.1K27M is most often associated with mutations in *ACVR1* (activin A receptor type 1) and *BCOR* (BCL6 corepressor) [5,40,44,45,46,47,48]. Although most studies of H3K27 mutations focus on its role in pHGG, mutations of H3K27 have also now been identified in adult cancers, including acute myeloid leukemia, melanoma, and glioma [8,13,49]

#### 2.1.2. Effects on K27 Methylation

H3K27 can be mono- (H3K27me1), di- (H3K27me2), or tri-methylated (H3K27me3), or acetylated (H3K27ac). The evolutionarily conserved Polycomb group (PcG) proteins are responsible for regulation of all three states of genomic H3K27 methylation. H3K27 is a target of the Polycomb Repressive Complex 2 (PRC2), which catalyzes methylation of this residue through the methyltransferase activity of the Enhancer of Zeste Homologue 1 or 2 (EZH1/2) components [50,51,52]. EZH1 and 2 proteins are members of the “SET” domain class, with the term SET being derived from Su(var)39, EZH2 (enhancer of Zeste), and Trithorax, three developmentally important genes that share a highly conserved S-adenosyl methionine (SAM)-dependent methyltransferase SET domain [53,54]. 

H3K27me2 and 3 at gene promoters is correlated with transcriptional repression, through the recruitment of PRC1 (Polycomb repressive complex 1), which is involved in chromatin compaction and catalysis of H2AK119 monoubiquitination, which inhibits transcriptional elongation [55]. H3K27me1 is less well-understood, with some reports suggesting a role in transcriptional activation [56,57]. In contrast, acetylation of H3K27 is well-known to be associated with active transcription, being highly enriched at the promoter regions and enhancers of transcriptionally active genes [57,58].

The detection of high-frequency K27M mutations in pediatric glioblastoma has intensified the efforts for identifying the link between H3K27/PRC2-mediated modifications and cancer. EZH2 mutations and expression changes have been described which suggest EZH2 has either proto-oncogenic activity or tumor suppressor activity in different cancer contexts [59,60,61,62,63,64]. The K27M mutation in either H3.1 or H3.3 plays a dominant role, leading to a global decrease in H3K27me2 and H3K27me3. This occurs even when the mutant protein comprises only 3%–17% of the total H3 population [65,66,67,68]. While H3.1 and H3.3 K27M mutant cancers both exhibit a global decrease in H3K27me3, the detailed biochemical output shows distinct patterns based on whether H3.1 or H3.3 is mutated [46,69]. K27M mutation of H3.3 would be expected to yield more transcriptional dysregulation, due to the increased incorporation of H3.3 at the sites of high transcriptional activity, versus H3.1 or H3.2. Indeed, DNA methylation profiling and RNA-Seq profiles of DIPG tumor samples indicate different patterns of DNA methylation and RNA expression in H3.3 K27M mutants, when compared with H3.1 or H3.2 K27M mutant tumors [69]. A small increase in H3K27ac was observed in some H3K27M DIPG samples, while other marks related to transcriptional activation, such as H3K4me3 and H3K36me3, remained unchanged [65,66]. Additionally, H3K27M DIPGs exhibit DNA hypomethylation, which may aid in tumor development [67]. The observed dominant loss of H3K27me3 is specific to H3K27M and to a lesser extent H3K27I, a rare H3K27 mutation observed in some DIPGs. No other amino acid substitution at K27 causes a global decrease in H3K27me3 when expressed in stable cell lines [41,65]. More recent work has verified the direct linkage of H3K27M to the loss of global K27me3 in vivo, since knockdown of *H3F3A* in DIPG-derived cell lines reverses the global decrease in K27me3 caused by H3.3K27M [70]. 

#### 2.1.3. The Tethering and Sequestration Model

**Figure 2:**
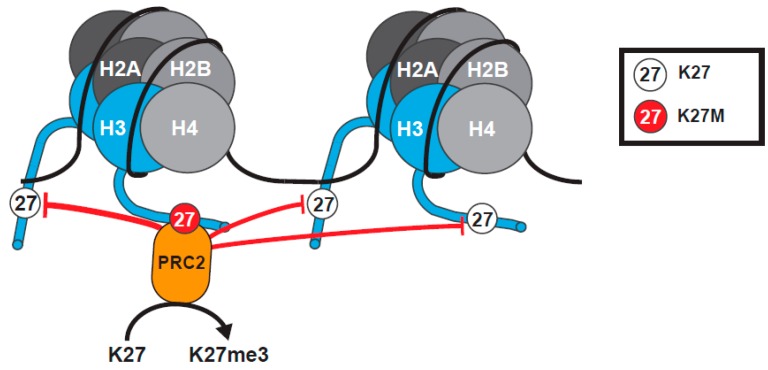
The tethering and sequestration model for K27M H3 Mutants. H3K27M (red) binds PRC2 with high affinity. The K27M mutation blocks methylation by PRC2 on K27 of the mutant histone tail, and sequesters PRC2, reducing global K27me3 levels within cells. This model is based on the assumption that levels of PRC2 are limited. Histone H3 tails have been truncated to amino acid 25 for clarity of presentation.

A model was proposed to explain how H3K27M mutation exerts dominant effects to reduce global levels of H3K27me3 (Figure 2). In this model, H3K27M stabilizes the binding of PRC2 to the mutant histone, sequestering the methyltransferase and preventing further deposition of H3K27me, leading to a global decrease in H3K27me3 [65,66,67,68]. Support for this model came from co-immunoprecipitation (Co-IP) data showing enhanced co-purification of H3K27M with PRC2, in comparison to Wild Type (WT) H3 [65,67], structural data indicating H3K27M binds to the SET domain of EZH2, and that the affinity of EZH2 for the H3K27M peptide is 16-fold higher than for its WT counterpart [71,72]. Further, the SET domain of EZH2 has a high affinity for binding to hydrophobic linear side chains, such as those of methionine or the non-canonical amino acid norleucine [65,72,73]. H3K27M peptides and nucleosomes can also inhibit PRC2 activity, similar to PRC2 inhibitors, by preventing the hydrolysis of SAM and the release of EZH2 [65,72]. 

However, not all of the data can be explained by the model. Genomic Chromatin Immunoprecipitation sequencing (ChIP-seq) revealed that some genes, such as strong PRC2 targets, actually retain normal levels of H3K27me3 in DIPG cells. A small subset even accumulate higher levels of the H3K27me2/3 mark and EZH2 [66,67,74,75]. Many of the genes exhibiting higher H3K27me2/3, which can span large chromatin regions, are cancer-associated and include cyclin-dependent kinase inhibitor 2A *(CDKN2A)* encoding the tumor suppressor p16/INK4A and cell cycle regulator Cyclin-dependent kinase 6 *(CDK6)* [66]. How some “islands” retain and even accumulate H3K27me3 in the midst of a global loss of H3K27me3 was not understood.

Additionally, some of the biochemical data does not fit the tethering and sequestration model. Herz et al. showed through Co-IP experiments that overexpressed H3.3 K27M does not exhibit an increased affinity for PRC2 [76]. In addition, in vitro nucleosome binding assays indicate that K27M containing nucleosomes bind PRC2, with an affinity similar to wild type nucleosomes [77]. Additionally, there is conflicting evidence on whether PRC2 components can actually localize to chromatin domains containing H3K27M, with some studies indicating that PRC2 is excluded from regions containing H3K27M [74,78,79]. In accordance with this, K27M nucleosomes are enriched for H3K27ac, supporting the exclusion of PRC2 from these loci [79]. Further complicating matters, there are cell-type specific differences in the effects produced by expression of K27M mutant H3 [75,80,81,82], and PRC2 cannot interact with K27M in all chromatin contexts, such as when the adjacent S28 residue is phosphorylated [73].

#### 2.1.4. Recent Advances with Understanding H3K27M

Although the full mechanism of how H3K27M contributes to disease remains unknown, several recently published studies have helped to reconcile conflicting data and have provided important new insights. 

The Helin lab generated a mouse model for DIPG through overexpression of platelet-derived growth factor B *(PDGFB)* and H3.3 K27M in mouse neural stem cells, which they then injected into the mouse pons [74]. Using these cells, whose transcriptional program resembles that of human DIPG, they showed that both the initiation of tumor development and its maintenance critically depend on *EZH2* function. Insight into how this occurs came from other studies showing repression of the *p16/INK4A* tumor suppressor in DIPGs harboring the H3K27M mutation [66,83]. Helin’s group found, similar to human DIPG cells, that high levels of H3K27me3 were retained at the promoter of *INK4A*, even though genome wide levels of H3K27me3 were decreased, and that perturbation of *EZH2* function alleviated *INK4A*-silencing, resulting in an expression of p16 and suppression of cell growth [74]. They confirmed their findings by deleting *INK4A* in human DIPG cells and showing that the cells were no longer sensitive to EZH2 inhibition. Further, ChIP-seq experiments revealed that the loci which retained H3K27me3 in K27M expressing cells tended to have high H3K27me3 levels in H3 WT cells, and correlated with strong Polycomb targets [74]. 

Many of the genes which have high levels of H3K27me3 are associated with CpG islands, which is intriguing, given the global hypomethylation observed in H3K27M DIPG [65,66,67,68,69,74]. While the decreased levels of DNA methylation can lead to genomic instability and transcriptional activation of normally silent regions, increased DNA methylation can silence normally transcriptionally active regions [84]. The Becher lab developed another murine model of DIPG with H3.3 K27M, activated *PDGFB*, and knock-out of *TP53*, which showed downregulated *p16/INK4A* expression with increased levels of H3K27me3 at the *p16/INK4A* promoter. In this system, inhibition of EZH2 decreased H3K27me3 to levels comparable to WT, but *p16/INK4A* expression could not be rescued [83]. DNA methylation of *p16/INK4A* promoter CpG islands can also suppress *p16/INK4A* expression, and treatment of these DIPG tumor cells with a DNA methyltransferase inhibitor restored *p16/INK4A* transcripts to WT levels, suggesting that DNA methylation plays a more important role than *EZH2* in regulation of *p16/INK4A* expression in these tumors [83,85]. The discrepancy between these two DIPG mouse models might be due to the cell type differences or differences in the mutational spectrum of each model, and it highlights the complexity of accurately modeling K27M-mediated DIPG. Further experiments in different K27M DIPG models are necessary to fully understand the role of DNA methylation and the retention/increase of H3K27me3 at specific loci to regulate gene expression in these cancers.

The Ren lab provide insight into the mechanism of how EZH2 is retained at some loci and lost from others, using live-cell single molecule tracking in mouse embryonic stem (ES) cells and in human DIPG cells [86]. They measured the search times and the residence times of several PRC2 and PRC1 components, and found that although cells expressing the H3.3K27M mutant or WT H3.3 have similar levels of EZH2 bound to chromatin, that in the presence of K27M, both the residency time of EZH2 on chromatin and the search time to find a specific target were increased around 1.5-fold [86]. This result supported the idea that PRC2 can be sequestered by its high affinity for H3K27M. Additional experiments monitoring binding kinetics, using just the catalytic lobe of EZH2, confirmed that the increased residency time relies on the interaction of the SET domain with K27M. The second finding, that K27M increases the search time for PRC2 to find its target, suggests that in K27M cells, the strength of the Polycomb binding site might differentially impact the binding of PRC2. So for example, high affinity Polycomb binding sites would be expected to compete better for binding PRC2 under conditions of reduced sampling efficiency. Such a model would fit nicely with the report that H3K27me3 and PRC2 are preferentially retained at high affinity Polycomb binding sites in H3K27M expressing cells [74].

**Figure 3:**
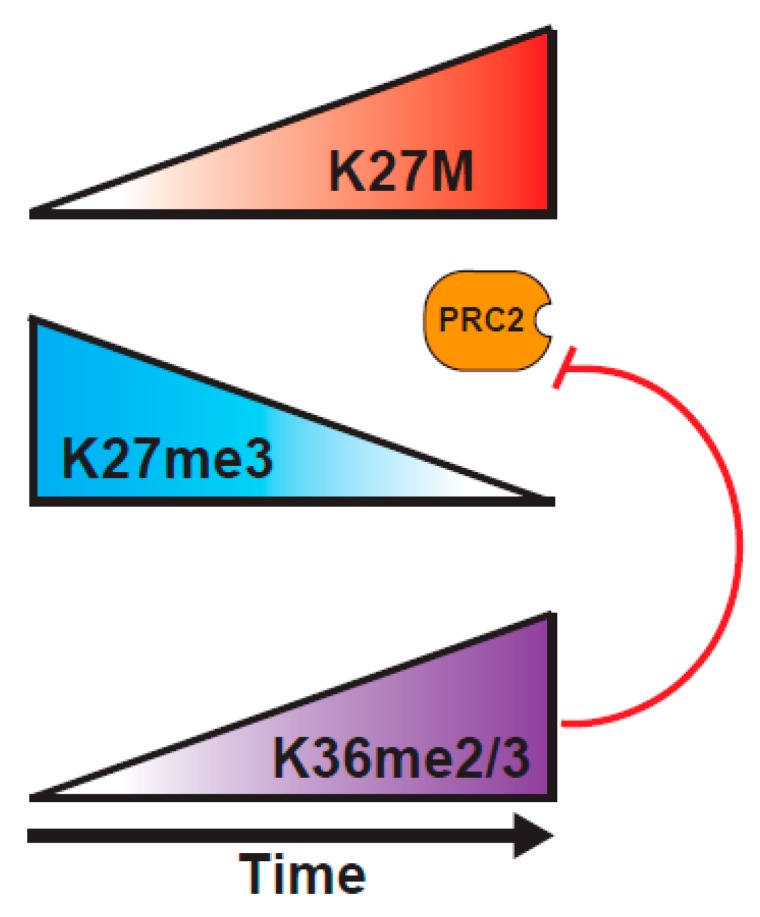
Revised views of K27M mutant H3 function. Induction of K27M recruits PRC2 to novel loci. K27M blocks PRC2 activity, causing global decrease in K27me3, with islands of retention of K27me3 at high affinity PRC2 recruitment sites. Transcriptional change on the loss of K27me3 promotes K36 methylation which further suppresses PRC2 activity [82]. Loss of K27me3 from bivalent promoters induces the expression of genes associated with neural development [75].

A study from the Reinberg lab probed the H3K27M mechanism of action, using a combination of biochemical approaches, and an inducible H3K27M expression system [82]. They tested a central tenet of the sequestration model, that there has to be sufficient H3K27M within the DIPG cells to bind most of the PRC2. Through careful mass spectrometric determination, they found that the cells vary widely in the concentration of PRC2 components, and that DIPG have relatively low levels of PRC2, such that K27M is at ~100-fold excess over PRC2 components, whereas K27M-engineered mouse ES cells express much higher levels of PRC2, so K27M is only at 10-fold excess over PRC2. This variance in abundance of PRC2 in different cell types might explain why some studies show much larger effects of the presence of K27M on H3K27me2/3 levels than others. Further, they demonstrated, using an inducible K27M expression system, that PRC2 can colocalize with H3K27M at novel sites that are not normally bound by PRC2 at early timepoints after induction of H3.3K27M, but that PRC2 is excluded from the regions containing K27M at later timepoints [82] (Figure 3). These results might help to explain the varying outcomes of experiments examining the colocalization of PRC2 and H3K27M in vivo, indicating that static experiments might not be well-suited to fully understanding the dynamic PRC2/H3K27M interaction. The question then arises as to why PRC2 is evicted from K27M loci at later timepoints. An interesting possibility would be through an increased deposition of H3K36me2/3 on H3K27M histones. Consistent with this model, K36me2 is the predominant modification found on H3.1 or H3.3 K27M tails, and the co-occurrence of K27M and K36me2 localization at 24 hours post-induction of K27M, when the EZH2 occupancy at K27M sites is lost, would fit with K36me2 preventing the EZH2 interaction with the H3 tail [82,87,88]. Such a model is reminiscent of recent findings showing the importance of the balance between PRC2 activity and the H3K36 modification in K36M mutant tumors that is discussed below [11].

Another intriguing finding from the Reinberg study [82] is the demonstration of a “memory” imparted to PRC2 from its previous association with K27M. Interaction of PRC2 with H3K27M nucleosomes causes a lasting decrease in the activity of the complex, possibly by causing a defect in the SAM turnover, or a defect in the spreading of the H3K27me3 mark [82]. Alternately, K27M interaction might inhibit the EZH2 automethylation (a modification of EZH2 shown to increase its histone methyltransferase activity) and prevent a conformational change required for EZH2 activation [89,90]. Such models align with the observed increase in search time for PRC2 to bind its target sequences in K27M expressing cells [86]. 

K27M might also contribute to disease through alterations of the post-translational landscape beyond H3K27me3 and H3K36me2/3. Early studies reported an increase in H3K27ac [65,76] and this observation was confirmed and extended in the assays of genomic distribution [75,79]. Surprisingly Stafford et al. found that the most pronounced acetylation difference between H3K27M mutant and H3 wild type DIPGs was a 20% increase in histone H4 acetylation [82]. The Baker lab developed a third mouse model for DIPG, using an inducible knock-in H3.3 K27M *H3F3A* allele, in lines deleted for *TP53* and expressing an activated *PDGFRA* [75]. Examination of K27M DIPG tumor cells compared with isogenic DIPGs that are WT for H3.3, revealed global changes in H3K27me3 and H3K27ac, but limited expression changes that were centered on genes involved in neural development. Specifically, the expression of genes with bivalent promoters that are marked by H3K27me3 and H3K4me3 in H3 WT cells is induced in K27M expressing cells, by loss of K27me3 [75]. These new mouse models provide the benefit of allowing a direct comparison of H3 WT and K27M tumors arising in the same region of the brain, where expression of the K27M mutant H3.3 protein is driven by the endogenous promoter. Further, there is a significant overlap between the gene expression profiles of these mouse DIPGs with human tumors [75]. It will be interesting to see if future analyses of this model confirm the importance of regulation of K36 methylation in tumor development, and the potential role of H4 acetylation changes in DIPG biology [82].

### 2.2. K36M

#### 2.2.1. Cancer Association of K36M

Mutation of H3.3 K36M was originally identified in chondroblastoma [7], a largely benign tumor of the epiphyseal plate cartilage that occurs in adolescents and young adults. In a sequencing survey of 70 patients, ~95% contained a mutation converting lysine 36 to methionine in H3.3, and 90% of the mutations were found in *H3F3B.* The H3.3 K36M mutant was the only recurrent mutation in these largely diploid tumors, and this finding, together with the low frequency of other genomic aberrations, suggests that the oncogenic driver in these tumors is the H3.3K36M mutation. Concurrent with the K36M discovery in chondroblastoma, some patients with more aggressive skeletal tissue tumors were identified that also exhibited K36M mutation of H3.3. These included 1% of conventional chondrosarcoma (mutated in *H3F3A*), and 7% clear cell chondrosarcoma (mutated in *H3F3B*) [7,91]. K36M mutations have also recently been identified in pediatric soft tissue sarcomas [11], as well as in a subset of head and neck squamous cell carcinoma (HNSCC) [12]. Interestingly, these HNSCC tumors do not share the bias towards mutation of H3.3, with only 2/11 mutant in H3.3 (*H3F3B*), and others predominantly mutant in different H3.1 encoding genes (8/11). Additionally, only H3.1 is mutated in the pediatric soft tissue sarcomas, although numbers reported to date are very low [11]. These findings reinforce that the K36M mutant likely functions in a dominant negative manner, since the presence of K36M on any histone H3 tail correlates with cancer, irrespective of which H3 protein subtype harbors the mutation.

#### 2.2.2. Effects on H3 K36 Methylation

Several recent studies have addressed the mechanism of action of the K36M mutant [11,65,92,93,94]. Although details of the reports differ, all have found that H3K36M peptides or nucleosomes more efficiently pull down the SETD2 H3 K36 methyltransferase or its fission yeast homolog, Set2, from cellular extracts than the wild type H3 controls [11,93,94], and that the K36M mutant dominantly inhibits the activity of SETD2, likely through this enhanced binding and sequestration of enzyme activity. This effect can be visualized on heterotypic K36M nucleosomes, where K36me2/3 is lost from the WT H3 protein [11]. Loss of SETD2 activity reduces global H3K36me3 since SETD2 is the only enzyme capable of tri-methylation of K36. K36M cells also have reduced K36me2, in part from SETD2 inhibition, but also from the inhibition of some but not all other K36 methyltransferases [11,93]. 

#### 2.2.3. Structural Considerations for K36M

Structural details for SETD2 bound to an H3 K36M peptide revealed that binding of the SET domain to the K36M peptide induces a large conformational change [92,94], but whether this change similarly occurs on binding to a wild type H3 peptide is not known, since that interaction is too unstable to capture. Proper folding of catalytically active SET domains requires the methyl-donor SAM, and this requirement holds also for formation of complexes of K9M and K27M H3 mutant peptides with their respective SET-domain methyltransferases [95]. In contrast, the structure of the K36M peptide in complex with SETD2 could be obtained in the presence of SAM or the methylation inhibitor *S*-adenosyl-l-homocysteine (SAH) [94]. Thus, although there are considerable parallels between the residues involved in the interface between the SET domains of different methyltransferases and their respective K to M mutant histone peptides (K9M, K27M, K36M), the fine details of how H3K36M associates with SETD2 differ. Determination of the structures of K36M peptides bound to SET domains of NSD1 and NSD2/MMS2 (which perform methylation upto and including K36me2) might shed light on why K36M inhibits NSD2/MMS2, but not NSD1.

#### 2.2.4. Biological Effects of K36M

Initial studies expressing low levels of exogenous H3.3 K36M in 293T cells showed decreased global levels of H3K36me2 and me3 [65]. Recent studies have extended these analyses by expressing K36M in more relevant cell types and by examining histone modifications in chondroblastoma tumor samples. These studies have confirmed the dominant negative effects of H3K36M on K36me2/3 in chondroblastoma [11,93]. To model the K36M mutation, Lu et al. took the approach of expressing exogenous K36 mutants in murine mesenchymal progenitor cells (MPC) at levels approximating those of the H3.3 K36M mutant in chrondroblastoma [11]. The MPC system is attractive, as different culture conditions can induce differentiation of different cell lineages (adipocytes, osteocytes as well as chondrocytes) from the progenitor cells. Early studies with this C3HT101/2 cell line showed that treatment of the cells with carcinogens impeded their differentiation and that mice implanted with carcinogen-treated MPCs developed undifferentiated sarcomas [96]. The finding that immunocompromised mice subcutaneously injected with MPCs that express K36M mutant, but not wild type H3.3, develop undifferentiated sarcomas, therefore further supports that the K36M mutant H3.3 drives oncogenesis [11]. Fang et al. took an alternate approach and generated knock-in cell lines with heterozygous H3.3 K36M mutant *H3F3B* in the T/C28a human chondrocyte progenitor cell line [93]. In this study, they compared gene expression and deposition of K36me2 and K36me3 in H3.3 K36M mutant chondrocytes with wild type cells, and hypothesized that DNA damage control was altered in the K36M cells [93]. Indeed they showed that colony formation was enhanced by K36M mutation, K36M mutants were less sensitive to staurosporine-mediated induction of apoptosis, and K36M mutant cells were defective for homologous recombination [93]. This latter result was consistent with studies of cells with defective SETD2 function [97].

#### 2.2.5. Interplay between K36M and K27 Methylation

**Figure 4:**
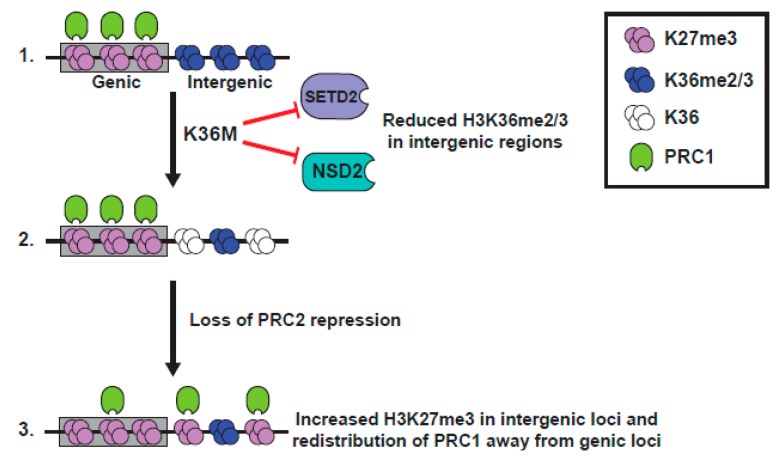
Model representing K36M mutant H3 function. Model derived from [11,93]. (1). K36M blocks function of SETD2 and NSD2, leading to reduction of K36me2/3. (2). In intergenic loci, reduction of H3K36me2 alleviates repression of PRC2, leading to increased intergenic K27me3. (3). Intergenic K27me3 recruits PRC1 away from genes, alleviating transcriptional silencing.

H3 K36 methylation has long been known as being antagonistic to the EZH2 function [87,88], and much of the Lu et al. paper is devoted to the analysis of the patterns of localization of PTMs in K36M expressing cells [11] (Figure 4). The Lu study documented a global increase in K27me3 in seven K36M chondroblastoma specimens, compared to an H3.3 WT chondroblastoma, using both western blot and quantitative mass spectroscopic analyses [11]. Although the study might have benefitted from the analysis of additional H3 wild type samples, the rarity of H3 WT chondroblastoma (5% of cases) made this difficult. Further, K27me3 was increased in MPCs that expressed a low level of exogenous H3.3 K36M, was increased in 293T cells that overexpressed K36M, and was increased in the WT H3 protein, in heterotypic K36M nucleosomes [11].

Importantly, the increase in K27me3 seen in K36M expressing MPC cells could be recapitulated by siRNA-mediated knockdown of three K36 methyltransferases, *NSD1*, *NSD2*, and *SETD2* [11], suggestive of a model whereby global loss of K36me2/3 promotes PRC2 activity to elevate K27me3. ChIP-seq of K36M expressing cells or K36 methyltransferase knockdown cells showed similar results, with levels of K27me3 unaltered on genes, but with a specific gain of K27me3 at intergenic regions that had previously lacked K27me3 and had been coated in K36me2/3. This redistribution of the relative levels of K27me3 at genes and the intergenic loci might cause a dilution of K27me3 (and importantly K27me3 “readers” or proteins that bind K27me3), away from the genic loci, resulting in a misexpression of genes normally silenced by PRC1. Indeed, there is a specific upregulation of PRC1-repressed genes in K36M expressing cells, and much of the transcriptional change seen in MPC cells expressing H3.3K36M is recapitulated by the knockdown of the RING1A/B proteins of the PRC1 complex that binds K27me3 [11]. Further, the block to differentiation of MPC cells caused by K36M expression is mirrored in the *RING1A/B* knockdown cells. Together, these data suggest a model wherein dilution of the repressive PRC1 complex, away from the genic loci in H3.3 K36M-expressing cells, leads to an altered gene expression and defects in differentiation, and highlights the importance of the dominant negative function of the K36M mutant in causing a loss of K36me2/3, as well as an enhanced K27 methylation, on wild type H3.

In contrast, Fang et al. found no change in K27me3 by a western blot analysis of their K36M knock-in cell lines, compared with the isogenic WT H3 cells [93]. The reason for this difference could simply be differences in the expression level of the K36M mutant allele in the different cell models, with a minimal level of K36M required for the dominant negative effects on K36 methylation, to promote K27 methylation changes. Alternately, the differences in K27me3 could be connected to differences in the relative expression of different K36 methyltransferases in the different cell types examined, since of the many K36 methyltransferases [98], only MMSET/NSD2 and SETD2 are known to be inhibited by K36M mutation [11,92,93,94]. Unfortunately, this study did not assess K27me3 in patient chondroblastoma samples [93]. 

## 3. The H3 G34 mutants

### 3.1. Cancer Association

Somatic mutations at G34 were first identified in *H3F3A* in pHGG, in the tumors of the cerebral cortex. Approximately 18% of the cortical pHGGs bear a G34R mutation or the less frequent G34V mutation in *H3F3A*, coding for H3.3. The reasons underlying *H3F3A* being selected over *H3F3B* for mutation, for G34V/R mutation being exclusive to H3.3, and for R being a more common mutant than V, are not understood, since the G34 codon is identical in both *H3F3A* and *H3F3B* genes, and a single base substitution in H3.3 or H3.1 encoding genes yields either V or R mutations [14]. Tumors bearing G34R/V mutations are also frequently mutated for *ATRX/DAXX* and *TP53* [5]. 

A different set of G34 substitutions to tryptophan (G34W), or more rarely leucine (G34L), were identified at high frequency (>90%) in H3.3 encoded by *H3F3A*, in giant cell tumors of bone (GCTB) [7]. These are locally aggressive, but rarely metastatic, benign tumors of young adults, which can cause extensive bone destruction [99]. Follow-up studies have now identified G34W mutations in a cancer syndrome that includes pheochromocytomas, paragangliomas, and GCTB [100]. Interestingly, G34W mutation in this syndrome is thought to arise post-zygotically, rather than as a somatic mutation, as distinct tumor types within the same individual have the G34W mutation, but their germline *H3F3A* is wild type. 

### 3.2. Structural Considerations for G34 Mutants

Several studies of SETD2 bound to H3 peptides have indicated that replacement of H3G34 with any other amino acid residue blocks SETD2 binding. This is because G33 and G34 are completely buried within SETD2, and G34 lies within a very narrow channel flanked by Phe 1668 and Tyr 1671 of SETD2, which cannot accommodate the presence of a larger amino acid at position 34 [92,94,101]. There is a considerable sequence homology with other SET domain methyltransferases at this region [92,94], but interestingly sequence variants at the two residues thought to be critical for restricting the size of the G34 channel in SETD2, would suggest that the channel is perhaps larger in the other methyltransferases, as shorter side chains occupy both positions in NSD1, 2, and 3 (Leu replaces both Phe and Tyr at the homologous positions). This might help explain why the G34 mutants do not completely block the activity of other K36 methyltransferases, since mass spectroscopy showed the H3.3G34R/V mutant histones retain monomethylation and some dimethylation of K36 [65]. However, molecular modeling of these leucine substitutions (to mimic NSD1, 2, or 3) into the SETD2 structure obtained in complex with K36M, suggests that there is still a steric clash with G34 substitutions [92]. 

The structure is also available for another enzyme that modulates methylation at H3K36, the demethylase KDM2A [102], which demethylates K36me2 and K36me1, but is not active on K36me3. Similar to SETD2, the G33-G34 region of the H3 tail occupies a narrow channel in KDM2A and substitutions of G34 are predicted to reduce binding to H3.

### 3.3. Effects on H3K36 Methylation in G34 Mutant Backgrounds

**Figure 5:**
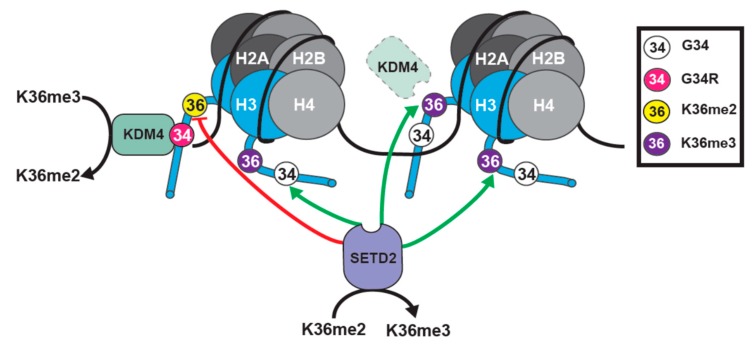
Model representing H3.3 G34R function. (1). G34R blocks SETD2 activity on the mutant histone tail. SETD2 activity on other H3 tails is unaffected. (2). KDM4 is sequestered by G34R mutant H3, enhancing K36me3 demethylation to K36me2 on the mutant tail. Further, loss of KDM4 from its normal targets (dashed line KDM4) leads to the accumulation of K36me3 at KDM4 target loci in G34R cells [105].

G34 lies towards the base of the H3 tail, close to the DNA entry and exit points of the nucleosome. G34 sits just 2 residues away from K36 and 4 residues from P38, a residue that can adopt distinct conformations to control K36 methylation [103]. G34R and V mutants have been shown to diminish H3K36me2/3 in cis on the same histone H3 tail, but exhibit no dominant effect to block K36 methylation on WT H3 tails [65] (Figure 5). G34W similarly reduces H3K36me3 [104]. How the G34 mutants exert dominant effects on histone H3 biology, remains unknown.

### 3.4. Biological Effects of G34 Mutants

#### 3.4.1. G34V

An early study of an H3.3 G34V mutant patient-derived pediatric high-grade glioma cell line, KNS42, compared with an H3 WT glioma cell line, revealed that H3K36me3 was differentially bound at ~160 genes, and, consistent with the role of K36me3 in transcriptional elongation, RNA polymerase II was co-recruited to these genes, and their expression was elevated [106]. These genes included several transcriptional regulators involved in neuronal differentiation and cellular proliferation, with the most differentially expressed gene being *MYCN* [106]. The upregulation of *MYCN* expression was recapitulated by transduction of H3.3G34V into normal human astrocytes and fetal glial cell lines, suggesting that the *H3F3A* G34V mutation enhances the expression of this oncogene, and that targeting MYCN stability might provide a therapeutic option for the treatment of H3.3 G34 mutant glioma [106]. These experiments indicate that although G34V reduces K36me2/3 only on the mutant H3 tail, transcriptional deregulation can be widespread. 

#### 3.4.2. G34W/L

Primary cell lines from GCTB have been derived, and study of these cells, together with isogenic knock-in *H3F3A* G34W and WT cell lines, has revealed that the H3.3 G34W promotes cellular proliferation [107]. Lim et al. showed that G34W expression induces transcriptional deregulation, along with splicing alterations, leading to frequent exon inclusion, which might cause an aberrant transcript stability, extension of open reading frames, and usage of alternative start sites to promote cellular growth [107]. A subsequent report that focused on the effect of H3.3 G34W/L substitutions [105] demonstrated that these mutants were defective for SETD2 mediated methylation in vitro. Stable expression of these mutants, at levels similar to endogenous H3.3 in HeLa cells, resulted in chromatin changes at the site of their deposition, with some reduction in H3K36me3 levels, but most noticeably elevation in H3K27me3, and some reduction in both H3K27ac and H3K9me3. The authors went on to demonstrate in vitro that the mutant histone tails did not affect PRC2 or p300 activity for methylation or acetylation of K27, respectively, suggesting that the effects on K27 modification were indirect effects of mutations [104]. These results suggest that similar to the K36M expressing cells, where H3K36me2/3 levels are reduced globally and H3K27me3 is promoted [11], local effects of G34 mutations on K36 methylation promote K27 methylation on the mutant histone tail. Consistent with this, increased binding of proteins that associate with H3K27me3 (PRC2 and PRC1 complex subunits) and decreased binding of proteins that bind H3.3K36me3 (ZMYND11) was also observed on G34 mutant H3.3 nucleosomes. Further, and counterintuitive to their ChIP data showing reduction in H3K36me2 levels in the G34 mutant cells, they found increased levels of the H3K36 methyltransferases NSD1, 2, and 3, bound to G34 mutant nucleosomes [104]. However, we point out that great caution needs to be taken when using antibodies to examine chromatin states of mutant histones. It is critical to assess, using peptide controls, whether antibodies can efficiently recognize particular marks in the context of a mutated histone tail. Alternately, antibody studies need to be coupled with a careful mass spectroscopy [108]. For example, our lab has demonstrated that recognition of H3K36me2 by two popular antibodies was diminished ~10-fold by G34R mutation [109]. If G34W similarly affects antibody recognition, H3K36me2 might be maintained or elevated on the mutant chromatin, more consistent with enhanced binding of NSD1-3 [104].

#### 3.4.3. G34R

Another study interrogated the role of the histone G34R mutant in a context where it provided the sole source of histone H3 in the cell, facilitating the analysis of possible biological effects of the mutation. Yadav et al. modeled the G34R mutant in fission yeast and found a drop in both H3K36me3 and H3K36ac levels, while H3K36me2 accumulated on the mutant tail [109]. H3K27 methylation was not analyzed, as this mark does not exist in fission yeast, which lack the PRC2 complex. Further, H3G34R mutation caused a defect in response to the S-phase induced DNA damage, a defect in homologous recombination–mediated DNA damage repair, as well as genomic instability. It is not currently know which phenotypes are linked to which chromatin changes in this system, or if indeed such causal relationships can be established, since in fission yeast a single enzyme Set2 mediates all three levels of H3K36 methylation [110], making it difficult to tease out the effect of loss of just K36me3. Whilst the reduction in H3K36me3 is consistent with mammalian studies showing G34R-disrupts SETD2-mediated K36 tri-methylation, the accumulation of H3K36me2 indicates Set2 can still methylate the G34R mutant fission yeast H3 tail, and is consistent with ChIP studies showing that Set2 still associates with G34R mutant chromatin [109]. However, the reduction in K36me3 and accumulation of K36me2, suggests that either Set2-mediated di- to tri-methylation of H3K36 is blocked by G34R mutation, or alternately that an H3K36me3 demethylase is activated. Currently there are no structural studies of the fission yeast Set2 protein to help address whether G34R mutation affects the Set2 function, and there are no published in vitro methyltransferase assays on the mutant histone, using fission yeast Set2. 

Fang et al. [101] sought to address the effects of H3.3 G34R, V or D mutation in mammalian cells. They showed that all three mutants decreased H3K36me2 and 3 in vivo, on the mutant H3 tail, and affected the function of NSD1, NSD2, and SETD2, in vitro. This group had previously shown that the DNA mismatch repair protein MutSα/MSH6 is recruited by H3K36me3, and that depletion of K36me3 or abrogation of the K36me3-MSH6 interaction, led to a mutator phenotype, similar to that seen in cells with defects in mismatch repair genes [111]. They found that cells harboring H3G34R/V mutations showed a slight elevation in mutation frequency, consistent with a reduction of MSH6 bound to chromatin caused by both the reduction in K36me3 in H3G34R/V cells, and decreased affinity of MSH6 for binding the mutant H3 tail [101].

#### 3.4.4. Does G34R Provide a New Source of Arg Methylation on the H3 Tail?

Histones are subject to multiple arginine methylation reactions [112], and we questioned whether the G34R mutant might provide a new site for modification on the histone tail. We queried whether R34 was subject to arginine methylation in fission yeast, and whether this might affect the modification of K36, since tri-methylation and acetylation of K36 are both reduced in H3G34R mutant backgrounds [109]. This idea was appealing, since in humans and budding yeast, R2 of histone H3 can be asymmetrically di-methylated by PRMT6 or Hmt1, respectively, and this modification reduces methylation at K4, which is similarly situated 2 residues away from the arginine [113,114,115]. Hmt1 targets RGG motifs usually [116], but can also mono-methylate Rrp43, and the sequence surrounding the mono-methylation site in Rrp43 is identical to that flanking H3G34R [117] (Figure S1a). Fission yeast have three arginine methyltransferases, Rmt1, Rmt3, and Rmt5 [118]. Rmt1 is homologous to the budding yeast and human Hmt1 and PRMT1, respectively, which are responsible for 80% of arginine methyltransferase activity [119,120,121]. To determine if G34R can be methylated, we performed in vitro methyltransferase assays, using recombinant Rmt1, ^3^H labeled SAM and H3-WT, and H3-G34R peptides, as targets. Rmt1 promoted incorporation of ^3^H on titration of G34R but not H3-WT substrate (Figure S1b,c), and mass spectroscopic analysis identified mono-methylation on G34R, demonstrating that Rmt1 can mono-methylate H3G34R (Figure S1d). However, the reaction was inefficient, as only 1% of the G34R peptide was methylated under conditions of enzyme excess (Figure S1e). To determine whether methylation of G34R occurs in vivo, we subjected semi-purified acid extracted histones from asynchronously growing yeast, to proteolytic cleavage and mass spectroscopy, but found no evidence for methylation at G34R, under conditions where we could detect 0.1% of signal (Figure S2). Thus, we concluded that fission yeast H3-G34R is not subject to arginine methylation in vivo. Whether H3.3G34R is methylated in pediatric cortical high-grade gliomas is an open question.

#### 3.4.5. A Question of Dominance for G34R Mutants

**Figure 6:**
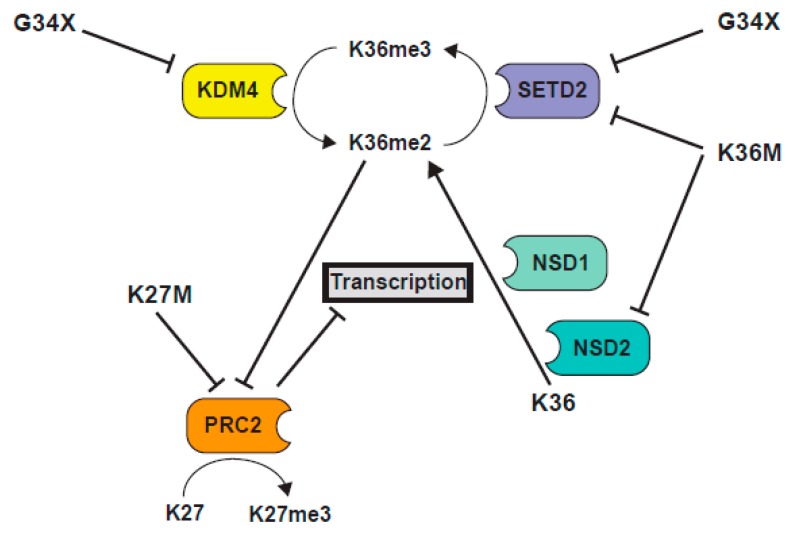
Schematic illustration of the interplay between K36 and K27 methylation pathways in histone mutant cells.

One of the biggest unknowns in the field of G34 mutant H3 biology is the nature of the dominant effect. A recent study sought to shed light on this issue [105]. Voon et al. targeted a *H3F3A* G34R mutation in mouse ES cells and found that it triggered a gain in H3K36me3 at some sites in the genome, although the overall levels of H3K36me3 measured by western blot analysis were unaltered [105]. They proposed that the elevation in K36me3 was caused by a suppression of activity of the KDM4 family of H3K36me3 demethylases, when bound to the G34R mutant H3.3 tail, similar to early models for K27M-mediated inhibition of PRC2 (Figure 6). In support, both the transcriptional and K36 tri-methylation profiles of G34R knock-in cells were similar to the KDM4-deficient cells, and they observed an association between the sites that are normally bound by KDM4 and regions of K36me3 accumulation in G34R cells. Further, since KDM4 also demethylates H3K9me3, they found that K9me3 levels increased in G34R cells, at sites that are normally bound by KDM4 proteins [105]. These results indicated a correlation between the sites of function of KDM4 and the loci altered by G34R mutation. However, the mechanism by which demethylase activity was suppressed by G34R mutation was less clear. One possibility was that G34R mutation reduced binding of KDM4 to H3.3. Consistent with this, in vitro demethylation assays using recombinant demethylases showed that the demethylase activity of KDM4A, B and C on K36me3 was suppressed by G34R [105], and structural studies indicated that G34R mutant H3.3 would bind KDM4 less strongly, due to difficulty in accommodating an amino acid larger than glycine, within the active site [122,123]. How a reduction in binding of KDM4 to G34R could lead to a dominant role for the G34R mutation is not clear. Surprisingly, Voon et al. show that KDM4 proteins bind more strongly to G34R, compared to wild type H3.3, when transfected KDM4 is immunopurified from cells expressing exogenous histones [105]. This data supports the sequestration model, wherein KDM4 proteins are tethered at sites of H3.3G34R deposition. A prediction from this model, which their data supports, is that in G34R cells, reduction of KDM4 (activity) at its normal targets, leads to an accumulation of K36me3 and trans-effects on the chromatin landscape. A second, untested, prediction is that the accumulation of KDM4 proteins at sites of G34R deposition, would reduce H3K36me3 at those sites. Accumulation of KDM4 might, therefore, work in conjunction with reduced SETD2 function at loci containing H3.3G34R, to ensure a robust loss of K36me3. Further examination of these mouse ES knock-in cells should prove fruitful, since this model circumvents the problems of the additional mutation of *TP53* or *ATRX*, which is predominant in G34R/V mutant tumors. In addition, the upregulation of H3K36me3 at particular loci in these ES cells is reminiscent of the upregulation of H3K36me3 at particular sites in the H3.3 G34V pediatric glioma cell line KNS42 [106]. It will be interesting to determine if genes that are upregulated and are enriched in H3K36me3 in KNS42 cells, are usually targeted by KDM4 demethylases.

## 4. Histone Genes Are Frequently Mutated in Adult as well as Pediatric Cancers

Here, we have attempted to summarize recent work on the most studied histone mutants that occur predominantly in pediatric and young adult patients, and to identify the common themes originating from the study of distinct mutants. To date, the best known oncohistones show clustering of mutations at, or in close proximity to, sites of PTM in the tail of H3 proteins and arise mainly in pediatric cancers. Recurrent histone mutations are, however, now being identified in a more diverse spectrum of cancers, including many adult cancers, and are found in histones other than H3, and in regions other than the histone tails.

The WHO recently designated H3 K27M mutant gliomas as a histologically heterogeneous, but midline-restricted series of cancers [43]. Several studies have now identified and characterized H3K27M mutant adult glioma [124,125,126,127,128,129]. K27M mutation is present in 35%–53% of adult midline gliomas that have wild type isocitrate dehydrogenase (IDH) [124,125,126,127]. An analysis of 21 such adult *IDH1* wild type H3K27M midline diffuse gliomas, revealed a median age of diagnosis of 32 years (range of 18–82 years), and that similar to pediatric K27M mutant tumors, the tumors were wild type for *BRAF*, were not amplified for *EGFR*, and showed infrequent *MGMT* promoter methylation [124]. Another study additionally found association of K27M adult gliomas with p53 overexpression, *ATRX* loss, and monosomy for Chromosome 10 [129]. Whether additional genomic perturbations that are common in pediatric K27M mutant tumors, such as amplification of *PDGFRA*, *CDK4/6*, or *MYC,* occur in adult K27M mutant gliomas, is not yet known. Interestingly, the specific location of the gliomas on the midline, varies with age. In children, K27M mutant gliomas are frequently located in the pons, whereas in adults, they are more frequent in the thalamus and spine [129].

Recurrent histone H3 mutations have also been identified in adult leukemia. H3K27M and K27I mutations have been identified in adult Acute Myeloid Leukemia (AML), where they associate with and collaborate with *RUNX1* mutations and translocations, to promote transformation [8]. The frequency of these mutations in AML is low (~0.5% of patients), but the mutation always occurs in a gene encoding H3.1 and always in association with *RUNX1* mutation or translocation [8]. A distinct set of histone mutations have been identified in both pediatric and adult T-cell acute lymphoblastic leukemia (T-ALL) [4,130]. Here, ~3% of samples sequenced showed H3K27 or K36 mutation to R, and less frequently K27 mutation to N [130]. K27N and K27T mutants have also been observed in a distinct study of T-ALL [131].

H2A and H2B mutations have been identified at high frequency in uterine and ovarian serous carcinomas in adult [132]. Although mutations in H2A and H2B are significantly enriched in these tumors (21% in serous cancers, compared with 5% in carcinomas from the same tissue of origin), only 1 particular mutation was identified twice (E57Q in *HIST1H2AB* which encodes H2A). These mutants exemplify the problems we now face in determining whether histone mutations identified in cancer are passenger mutations or have functional significance. In this case, 10 mutants were clustered within 5 of the 37 genes that encode H2A and H2B, and the absence of mutants in normal tissue of the same patients allowed confirmation that the mutants were somatic, and not caused by single nucleotide polymorphisms. This information, together with evidence for tumor-specific amplification of a segment of chromosome 6 on which many of the genes that are mutated are clustered, contributed to the belief that these unique mutants might be of significance. Functional studies, in fact, demonstrated that the mutants might contribute to epithelial-mesenchymal transition and the evolution of carcinoma to sarcoma [132]. 

A recent study from the Allis lab highlights the previously unappreciated wide spectrum and frequency of histone mutants in cancer [13]. They also provide an extremely useful resource- the Oncohistone dataset- (available through the cBioPortal https://bit.ly/2GXH5Ve) which provides an interactive interface and catalogs over 2500 cancer samples from 98 studies that carry histone mutations [13]. The authors suggest that as a conservative estimate, somatic mutations in histones occur in 4% of cancers [13]. Given the caveats to identification of histone mutations outlined below, the actual levels of mutation could be much higher, since these mutations can occur in different genes coding for the same histone, which reduces their “apparent” frequency. Earlier sequencing efforts might have overlooked these mutations as noise, especially for the canonical histones, as there is so much redundancy in genes that code for particular proteins. Further, sequencing of cDNA might have selected against identification of mutations in canonical histones which are encoded by non-polyadenylated transcripts.

## 5. Conclusions and the Future for Histone Mutant Research in Cancer

Since their discovery in 2012, the occurrence of high frequency mutations in histone H3 proteins in cancer has provoked an avalanche of research interest in histone biology. How the mutation of a single allele, of thirty that code for H3 isoforms, can dominantly influence the chromatin landscape to promote oncogenesis, is the subject of much scientific debate. While great progress has been made in our understanding of select mutants, there are still many outstanding questions.

One outstanding question is why there is frequently a bias towards mutation of one histone H3 variant encoding gene, in a particular tumor type. In the tumor samples to date, H3.3 encoding genes are predominantly targeted over H3.1 or H3.2. This bias might partially be due to the nature of H3.3, since it can be deposited throughout the cell cycle and is enriched at regions of high transcriptional activity, and also in constitutively repressed domains. Consistent with this, the frequency of mutation of proteins involved with H3.3 deposition, such as ATRX, appears to be much higher than that seen for chaperones for H3.1 and H3.2 in pediatric cancer (pecan.stjude.cloud/proteinpaint) [133]. Why one particular H3.3 coding gene should be mutated more frequently than another, can rarely be explained by codon bias [14], but is more likely due to temporal and spatial differences in the expression of the genes during embryonic and post-natal development [36]. 

One key finding in the study of histone mutant tumors is the importance of cross-talk between different methylation marks on the H3 tail, in particular, the importance of regulation of methylation on K36me2/3 and K27me3. In pHGG, early studies focused on the effect of loss of K27 methylation in H3K27M mutant tumors. There is, however, a growing awareness of the importance of disruption of K36 methylation, and the enzymes involved in its deposition, such as SETD2, NSD1, and NSD2 in pHGG, where 15% of histone wild type cortical high grade gliomas bear deleterious mutations in SETD2 [134], and H3K36 methylation is affected by H3.3 G34R/V mutation in an additional ~18% of cortical gliomas. With new studies also showing altered H3K36 methylation patterns in H3K27M mutant glioma (which occur in the midline brain and brainstem and include 80% of DIPG), deregulation of the K36 methylation axis appears to now be a unifying theme in pHGG (Figure 5). Similarly, in K36M mutant chondroblastoma and head and neck squamous cell carcinoma, although loss of K36 methylation was perhaps an expected result, new data suggests that K36M reduces K36 methylation in the intergenic loci, to promote K27 methylation in these domains and redistribute repressive complexes away from Polycomb-repressed genic regions, resulting in a blockage in mesenchymal differentiation [11,93].

The challenge for the field now is to rapidly hone in on mutants that are likely to play a causal, rather than a passenger role in cancer, and to identify phenotypes for these mutants [13]. There are clear road maps for some of the newly identified mutants that have been laid out by decades of classical genetic and biochemical studies in yeasts and other “simple” models, where the copy number for histone genes is reduced, compared to humans. Such models provide exquisite tools for understanding histone biology, especially in situations where the genetic landscape of tumors that bear histone mutations is complicated, with multiple drivers required for oncogenesis. Further, it is critical that researchers embrace the complexity of PTM landscape changes mediated by the histone mutants and fully document PTM changes, while cognizant of the limitations of antibody studies, and aim to couple their studies with highly refined mass spectroscopic approaches. It is also key to determine the primary and secondary effects of the histone mutations. New advances in development of tools for in vitro assessment of the roles of histone mutations, post-translational modifications [135], and the use of temporal control of mutant histone expression in cells, will be instrumental for a full understanding of histone biology and its contribution to cancer. 

## Supplementary Materials

The following are available online at https://www.mdpi.com/2072-6694/11/5/660/s1, Figure S1: A synthetic peptide carrying the G34R mutation is methylated by Rmt1 in vitro; Figure S2: Fission yeast H3 G34R is not arginine-methylated in vivo. Materials and Methods.
