# Supplementary material for: Histone H3 Mutations: An Updated View of Their Role in Chromatin Deregulation and Cancer
Source: Cancers (Basel). 2019 May 13;11(5):660. doi: 10.3390/cancers11050660 (PMC6562757; doi:10.3390/cancers11050660)
Supplement: Supplementary file 1 [file cancers-11-00660-s001.pdf]

Supplementary Materials

Review

Histone H3 Mutations: An Updated View of Their Role in Chromatin Deregulation and Cancer

Brandon R. Lowe, Lily A. Maxham, Joshua J. Hamey, Marc R. Wilkins and Janet F. Partridge

1. Plasmid DNA and recombinant proteins

Codon optimization was performed for expression of fission yeast proteins in *E.coli*. Codon optimized Rmt1 (*S. pombe*) with 1 x FLAG was cloned into pET28a plasmid with N-terminal 6 x HIS tag by GenScript to generate JP-2597. Fission yeast Histone H3 and mutant version (G34R) were codon optimized by use of codon utilization analyzer 2.0 for expression in *E.coli* and the cDNAs were synthesized by IDT and were cloned into pCDF duet (Novagen) in frame with an N-terminal 6 x HIS tag for bacterial expression (vectors JP-2395 (WT) and JP-2489 (G34R). Expression of *S. cerevisiae* Hmt1 was from vector pET15b (HMT1) [1].

Rmt1 and Hmt1 constructs were transformed into BL21 (DE3)-RIPL (Stratagene) chemically competent cells and grown overnight in a 100 mL starter culture. Three liters of culture were prepared for each construct by inoculating 10 mL of starter culture per liter of fresh LB media containing antibiotics and 8 g/L glucose. The cultures were shaken at 200 rpm at 37 °C and induced at 18 °C by 0.5 mM IPTG once the O.D. reached 0.7–0.8. After growing overnight, cells were harvested by centrifugation for 15 minutes in an F9-6x1000 rotor (Thermo Lynx 6000 centrifuge) at 3200 rcf. The cells were resuspended in lysis buffer (50 mM Tris 8.3; 250 mM NaCl; 10% glycerol; 2 mM beta-mercaptoethanol (BME); Roche Complete EDTA-free protease inhibitor tablets) and lysed using a microfluidizer. The soluble fraction was isolated after centrifugation in an F20-12x50 rotor (17,000 rpm in a Thermo Lynx 6000 centrifuge) for 30 minutes. Sodium chloride was added to a final concentration of 1M, and then nucleic acids were precipitated by adding 10% polyethylenimine/10% hydrochloric acid to a final concentration of 0.3%. This solution was centrifuged at 2,900 rcf for 15 minutes in a benchtop centrifuge, and the supernatant was collected. Ammonium sulfate was added to 70% saturation, and the precipitate was isolated by centrifugation for 30 minutes in an F20-12x50 rotor (Thermo Lynx 6000 centrifuge). The pellet was resuspended in buffer NBB (50 mM Tris 8.3; 500 mM NaCl; 25 mM Imidazole; 10% glycerol; 2mM BME) and bound batchwise to Qiagen Ni-NTA agarose. After 5 washings of the Ni-NTA agarose with NBB, the protein was eluted in a single step with 50 mM Tris 8.3; 500 mM NaCl; 10% glycerol; 2 mM BME; 250 mM imidazole. The eluted sample was then dialyzed overnight against 45 mM Hepes 7.6; 360 mM NaCl; 36 mM imidazole; 10% glycerol; and 18 mM BME. The sample was then mixed with 100% glycerol to achieve a final concentration of 50%. Proteins were quantified using a Nanodrop. Recombinant fission yeast histones were purified from *E. coli* by "The Histone Source" at Colorado State University following standard procedures for histone purification [2].

2. Histone purification for Mass spectrometric studies

Histones were purified from fission yeast following a previously described purification protocol with some modification [3]. A 150 mL culture was inoculated to a density of 1.4 $x10^6$ cells/mL in 4X YES media from a starter culture grown overnight in 4X YES at 25 °C. The cells were grown to a density of 3.6 $x10^7$ cells/mL and harvested by spinning at 3,000 RPM for five minutes. Cells were washed with H_2O containing 10 mM sodium butyrate, followed by NIB buffer (250 mM sucrose, 20 mM HEPES pH 7.5, 60 mM KCl, 15 mM NaCl, 5 mM MgCl2, 1 mM CaCl2, 0.8% Triton X 100, 0.5 mM spermine, 2.5 mM spermidine, 10 mM sodium butyrate, 1 mM PMSF, and Sigma yeast protease inhibitor). The pellet was frozen on dry ice and stored at −80 °C. For lysis, the pellet was resuspended in 2 mL of NIB and transferred to a 7 mL bead beater tube along with chilled acid washed glass beads.

The sample and beads were frozen on dry ice and bead beaten for a total of 10 minutes at max power. The sample was collected by "piggy backing" into a 50 mL Oak Ridge tube at 3,000 RPM at 4 °C in a benchtop centrifuge. The sample was then pelleted by centrifuging at 13,000 RPM for 10 minutes at 4 °C in a Beckman Avanti centrifuge J-30I centrifuge using a JA25.50 rotor. The supernatant was discarded and the pellet was washed in 15 mL of NIB. The pellet was then resuspended in 10 mL of 0.4 N H_2SO_4 and sonicated for one minute at max power before incubation for two hours on a rotating wheel at 4 °C. The sample was pelleted by centrifuging at 13,000 RPM at 4 °C for 10 minutes and the supernatant was transferred to a new tube along with 5 mL of 5% buffer G (5% guanidine HCl and 100 mM potassium phosphate buffer pH 6.8) where the pH was adjusted to 6.8 using 5N KOH. 0.5 mL of Bio-Rex™ pre-equilibrated in 5% buffer G was added to the sample and incubated at room temperature with rotation overnight. The resin was then washed 2 X with 20 mL of 5% buffer G and incubated with 3 mL of 40% buffer G (40% guanidine HCl and 100mM potassium phosphate buffer pH 6.8) for one hour at room temperature to elute the bound protein. Buffer exchange and concentration was performed against 5% acetonitrile with 0.1% TFA to a final volume of 150 µl and the sample was stored at −80 °C. Protein concentration was measured and 10 µg of sample was run on a gel for quality control.

3. Mass spectrometry for arginine methylation-LC MS/MS Analyses

50 ug of acid extracted histone samples were run on a 12% NuPAGE Bis-Tris polyacrylamide gel (Thermo Fisher Scientific) in MOPs running buffer and the gels were stained overnight using QC Colloidal Coomassie Stain (BioRad). Histone H3 bands were then excised from the gel and destained with 500 µL 50% acetonitrile/50% 50 mM ammonium bicarbonate (v/v) and then dehydrated with 500 µL acetonitrile. Proteins were then reduced with 500 µL 10 mM dithiothreitol for 1 hour at 37 °C, before alkylation with 500 µL 55 mM chloroacetamide for 1 hour at room temperature. Bands were dehydrated again with 1 mL acetonitrile before digestion with 100 ng rLysC (Promega, Fitchburg, WI) in 200 µL 50 mM ammonium bicarbonate for 18 hours at 37 °C. The supernatant was transferred to a new tube before peptides were extracted from gel bands with 200 µL 50% acetonitrile/50% 0.1% formic acid (v/v) for 30 minutes and then 200 µL acetonitrile for 10 minutes. Peptides were dried in a SpeedVac™ (Thermo Fisher Scientific, Waltham, MA, USA) before being resuspended in 20 µL 0.1% formic acid. Peptide assays of arginine methyltransferase activity (see below) were resuspended in 100 µL 0.5% heptafluorobutyric acid. 20 µL of this was then prepared for mass spectrometry using Bond Elut OMIX C18 pipette tips, 10—100 µL (Agilent, Santa Clara, CA, USA), according to the manufacturer's protocol. Two biological replicates were used for analysis.

LC-MS/MS

Samples were separated by nano-liquid chromatography (LC) on an UltiMate 3000 HPLC autosampler system, according to previous methods [4], before ionization by electrospray and analysis in a Q Exactive Plus (Thermo Fisher Scientific) mass spectrometer. Survey scans (350-1,750 *m/z*) were acquired in the Orbitrap (resolution = 70,000 at 200 *m/z*, automated gain control target = 1 × 10^6). Precursor ions were selected for fragmentation by data-dependent acquisition in combination with an inclusion list containing the *m/z* values of the methylated peptides of interest. For each round of MS2 analysis, up to ten ions were sequentially isolated and fragmented using HCD (normalized collision energy = 30, maximum injection time = 125 ms, automated gain control target = 1 × 10^5), before fragment ions were analyzed in the Orbitrap (resolution = 17,500). The most abundant precursor ions within 10 ppm of any *m/z* value on the inclusion list were preferentially fragmented. "If idle" was set as "pick others", allowing for any additional MS2 scans to be assigned to the most abundant ions not within 10 ppm of any *m/z* value on the inclusion list. Dynamic exclusion was enabled with an error tolerance of 10 ppm and an exclusion duration of 15 seconds.

Data from histone digests were analyzed with MaxQuant (v. 1.5.8.0) [5]. Data were searched against *S. pombe* proteins from the SwissProt database (07-2017), modified to include the G34R mutation in histone H3 (P09988 and P10651, residue 35), as well as contaminants. The following settings were used for searches: Enzyme: LysC; Max missed cleavages: 4; Variable modifications:

Methyl (KR), Dimethyl (KR), Trimethyl (K); Precursor ion tolerance: 4.5 ppm; Fragment ion tolerance: 20 ppm. Peptide-spectrum matches were filtered at a false discovery rate of 1%. Extracted ion chromatograms (XICs) of peptides and their methylated forms were obtained in Thermo Xcalibur Qual Browser 2.2 SP1.48 by taking mass windows (±10 ppm) of the relevant *m*/*z* for the monoisotopic peak, and applying a scan filter to only extract precursor scans.

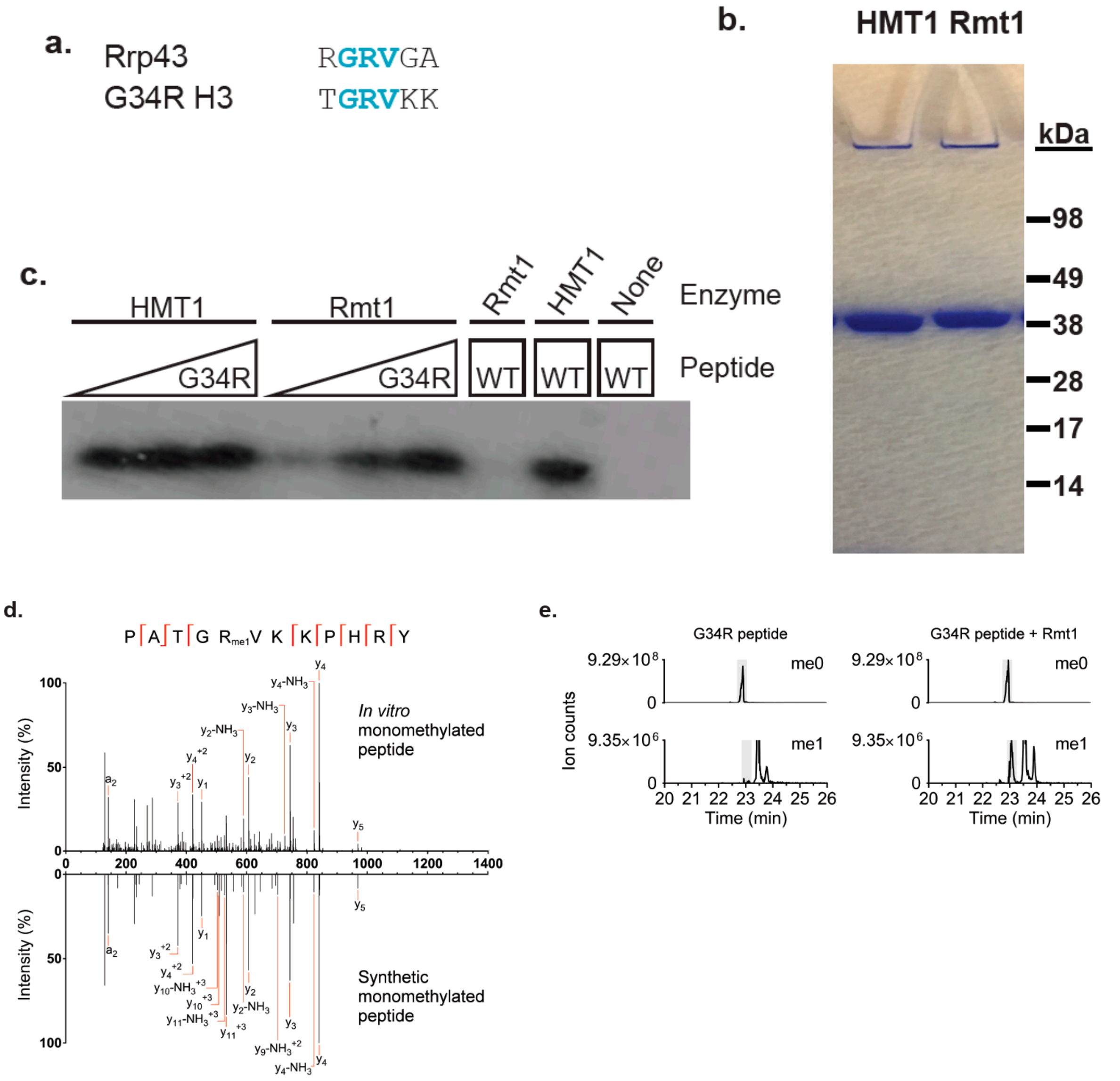

Figure S1. A synthetic peptide carrying the G34R mutation is methylated by Rmt1 *in vitro*. (**a**) Alignment of the HMT1 target sequence in Rrp43 with the H3 G34R sequence. (**b**) Coomassie stained gel of purified bacterially expressed HMT1 and Rmt1 proteins. (**c**) Fluorograph of in vitro ^{3}H SAM methylation assay. To determine whether G34R can be methylated, synthetic peptides for wild-type H3 (a.a.30-41) or H3 peptide carrying the G34R mutation (PATGRVKKPHRY) were subject to *in vitro* methyltransferase assays using recombinant *S. cerevisae* HMT1 or *S. pombe* Rmt1 and ^{3}H labeled S-Adenosyl Methionine (SAM). Reaction products were subject to SDS PAGE and exposed to film. A dose response of incorporation of ^{3}H on titration of G34R peptide substrate was seen in the presence of Rmt1 (lanes 4-6), whereas HMT1 promoted incorporation of ^{3}H on both substrate peptides (lanes 1-3). (**d**) In vitro methylation reactions were performed on H3-G34R peptide with or without Rmt1 in the presence of AdoMet, before analysis by LC-MS/MS. MS/MS spectrum showing monomethylation of the G34R peptide when incubated with Rmt1 (top). The identity of this peptide was confirmed by comparison to the MS/MS spectrum of a synthetic peptide containing monomethylated R34 (PATGR$_{me1}$VKKPHRY, bottom). Dimethylation was not observed. **(e)** Extracted ion chromatograms (XICs) were taken of the un- and mono-methylated forms of the G34R peptide, revealing ~1%

monomethylation only when incubated with Rmt1 under conditions of enzyme excess, suggestive that the reaction is highly inefficient. XICs were obtained by taking mass windows (±10 ppm) corresponding to the *m/z* values of the un- and mono- methylated forms of the peptide (PATGRVKKPHRY^{+4}). Elution times of peptides are shaded. me0: unmethylated; me1: monomethylated.

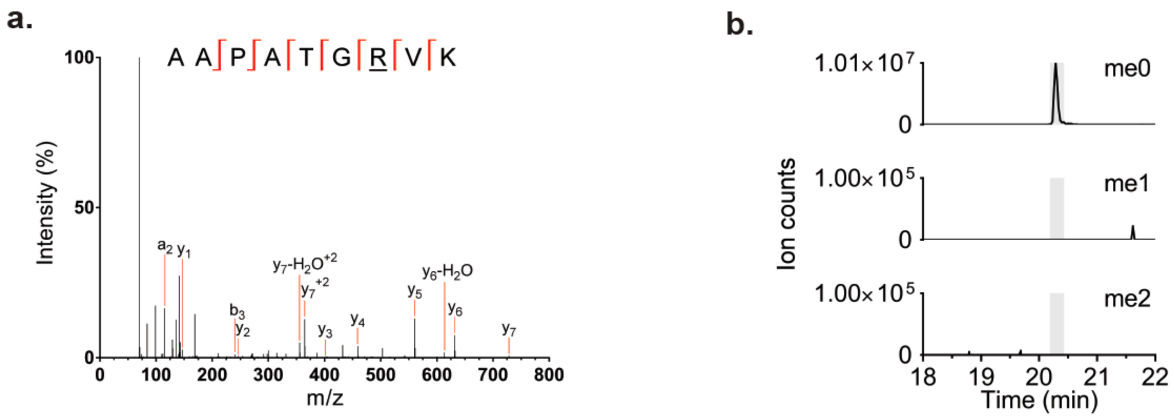

Figure S2. Fission yeast H3 G34R is not arginine-methylated *in vivo*. Histones purified from H3-G34R fission yeast were electrophoresed and H3 gel bands excised. Gel bands were digested with LysC and analysed by LC-MS/MS. (**a**) MS/MS spectrum identifying an unmethylated LysC peptide AAPATGRVK corresponding to residues 28-36 of histone H3. R34 is underlined. (**b**) Extracted ion chromatograms (XICs) of the peptide in (**a**) were taken for its un-, mono- and di-methylated forms, demonstrating that only the unmethylated peptide is present. This indicates that R34 is completely unmethylated *in vivo*. XICs were obtained by taking mass windows (±10 ppm) corresponding to the *m/z* values of the un-, mono- and di-methylated forms of the peptide (AAPATGRVK^{+2}). Elution times of peptides are shaded. me0: unmethylated; me1: monomethylated; me2: dimethylated.

References

1. Hart-Smith, G.; Low, J.K.; Erce, M.A.; Wilkins, M.R. Enhanced methylarginine characterization by post-translational modification-specific targeted data acquisition and electron-transfer dissociation mass spectrometry. *J. Am. Soc. Mass Spectrom.* **2012**, *23*, 1376–1389, doi:10.1007/s13361-012-0417-8.
2. Dyer, P. N.; Edayathumangalam, R.S.; White, C.L.; Bao, Y.; Chakravarthy, S.; Muthurajan, U.M.; Luger, K. Reconstitution of nucleosome core particles from recombinant histones and DNA. *Methods Enzymol.* **2004**, *375*, 23–44, doi:10.1016/S0076-6879(03)75002-2.
3. Sinha, I.; Buchanan, L.; Ronnerblad, M.; Bonilla, C.; Durand-Dubief, M.; Shevchenko, A.; Grunstein, M.; Stewart, A. F.; Ekwall, K. Genome-wide mapping of histone modifications and mass spectrometry reveal H4 acetylation bias and H3K36 methylation at gene promoters in fission yeast. *Epigenomics* **2010**, *2*, 377–393, doi:10.2217/epi.10.18.
4. Hart-Smith, G.; Raftery, M.J. Detection and characterization of low abundance glycopeptides via higher-energy C-trap dissociation and orbitrap mass analysis. *J. Am. Soc. Mass Spectrom.* **2012**, *23*, 124–140, doi:10.1007/s13361-011-0273-y.
5. Tyanova, S.; Temu, T.; Cox, J. The MaxQuant computational platform for mass spectrometry-based shotgun proteomics. *Nat. Protoc.* **2016**, *11*, 2301–2319, doi:10.1038/nprot.2016.136.
